# Analysis of readability of the top web searches for pediatric inborn errors of fatty acid metabolism

**DOI:** 10.1016/j.ymgmr.2025.101195

**Published:** 2025-01-25

**Authors:** Katelyn Sawyer, William Miller, Courtney Popp, Chloe Strege, Cindy Eide, Jakub Tolar

**Affiliations:** a Department of Pediatrics, Division of Blood and Marrow Transplantation and Cellular Therapies, University of Minnesota, Minneapolis, MN, United States of America; b University of Minnesota Medical School, Minneapolis, MN, United States of America

## Abstract

**Background:**

Disorders of fatty acid oxidation (FAOD) are estimated to account for around 1 in 10,000 live births, and with modern newborn screens, these conditions are often identified in childhood. However, not all parents will receive regular medical follow-up, and varying levels of parental health literacy can influence their reliance on online resources for information. Therefore, assessing the readability of online materials is critical to ensuring accessible and comprehensible patient education. Understanding the readability landscape informs our efforts to improve the quality of online resources and to support parents and patients in navigating the diagnosis of an FAOD.

**Objective:**

Our goal was to evaluate the readability of public facing online materials concerning the 10 most common disorders of fatty acid oxidation, with consideration given to the recommended reading levels by the National Institutes of Health (NIH) and the American Medical Association (AMA).

**Methods:**

Using Flesch-Kincaid, Coleman-Liau, and SMOG readability indices, we analyzed the top 25 internet search results for each disorder. Excluding empty or paywalled content, 232 publicly accessible materials were assessed.

**Results:**

Mean readability ranged from 11.64 to 12.85, indicating generally higher complexity than recommended. Only 15.5 % of materials met NIH's 8th grade reading level guideline, and 3.9 % met AMA's 6th grade level. Variability existed between disorders, with percentages meeting guidelines ranging from 0 % to 25 % for NIH and 0 % to 8.3 % for AMA.

**Conclusion:**

Ensuring readability of online resources for rare disorders of fatty acid oxidation is crucial, particularly given the prevalence of childhood diagnosis and varying levels of parental health literacy. Parents may rely on easily accessible but potentially complex materials found through online searches, highlighting the importance of aligning online content with recommended reading levels. Improving readability can enhance accessibility and understanding and facilitate informed decision-making and optimal care for patients.

## 1 Introduction

Health literacy is an important social determinant that influences individuals' ability to access, understand, and utilize health information effectively. Low health literacy has been associated with several negative outcomes, including increased hospitalizations, greater utilization of emergency care, and lower uptake of preventative measures. [[1], [2], [3]] While the mechanisms underlying this relationship are complex and multifaceted, one contributing factor is likely the communication barrier between patients and medical professionals. There is a stark difference between the reading level of clinicians and the general population: the average adult in the United States reads at an 8th grade level, and nearly one fifth of adults in the United States read at or below a fourth grade level. [4]

In clinical settings, healthcare jargon and the complexity of medical information pose additional challenges, particularly for individuals with limited health literacy. As a result, patients often seek supplemental health information online, emphasizing the importance of assessing the readability of online materials. [5] Understanding the readability landscape of online resources is crucial for ensuring accessible and comprehensible patient education. Measuring the readability of text can be assessed using validated formulas that assess the number of years of formal education (i.e. grade level) that a reader would be expected to need in order to be able to engage with and understand a text. [6] There are several such formulas, of which we selected three for use in our analysis: the Flesch Kincaid Grade Level (FKGL), the Coleman-Liau Index (CLI), and the Simple Measure of Gobbledygook (SMOG). [[7], [8], [9]]

Fatty acid oxidation disorders (FAOD) are estimated to affect around 1–2 in 10,000 live births and are often identified in childhood through modern newborn screening programs. [10] However, not all parents receive regular medical follow-up, leading to varying levels of reliance on online resources for information. Thus, evaluating the readability of online materials concerning the most common FAODs is essential for supporting parents and patients in navigating the diagnosis and management of these conditions. The aim of our study was to assess the readability of public-facing online materials related to 10 of the most common disorders of fatty acid oxidation.We hypothesized that if parents of a child recently diagnosed withFAOD were to search online for information on their child's disease, the results they find would largely be written at a level far above the national average reading level.

## 2 Methods

### 2.1 Study design

We examined the top 25 internet search results for 10 of the most common disorders of fatty acid oxidation. [10] Each search was conducted in an incognito browser, to avoid bias from our prior search history or user data, using the full written name of the disease, such as “Medium chain acyl-CoA dehydrogenase deficiency”. We excluded one FAOD that was commonly listed in the most common disorders of fatty acid oxidation, Long-chain Acyl-CoA Dehydrogenase Deficiency (LCADD), because of debate over whether this truly constitutes a unique disease in humans from the closely related Very Long Chain Acyl-CoA Dehydrogenase Deficiency (VLCADD), with which it also shared significant search result overlap. We elected not to limit our search only to patient facing materials or peer reviewed journals, under the assumption that parents may also not limit themselves to reading only patient facing materials or peer reviewed journal entries. We did, however, exclude any results that were locked behind a paywall, under the assumption that most parents would not pay for access to a medical journal or other resource and would instead move on to the next freely readable material available. Our initial search returned 250 unique resources, of which 18 were excluded due to requiring some form of payment for access, leaving 232 freely available online texts that were included in our analysis. Of these 103 were from freely accessible scientific journals, 15 were from hospitals or universities, 67 were from community resources, and 47 from government sponsored pages. All materials were recorded and organized by disease with accompanying URL.

### 2.2 Readability analysis

Each of our 232 selected materials were analyzed using the three previous listed readability indices: the Flesch Kincaid Grade Level (FKGL), the Coleman-Liau Index (CLI), and the Simple Measure of Gobbledygook (SMOG). These indices are well-validated and easily comparable, as they each report results as a grade level equivalent (i.e. the number of years of formal education needed to reasonably comprehend the material). Each formula calculates readability in different ways, placing different weights on variables such as average word length and average sentence length. [11] The advantages and disadvantages of each tool are outlined in Supplemental Table 1. [12] Because of these differences between indices, it is a best practice to analyze material using multiple different indices, as was done in our study. Analysis was conducted using an online readability scoring tool.

### 2.3 Statistical analysis

All statistical analyses, graph, and figure creation were performed using GraphPad Prism version 10.2.3, GraphPad Software, Boston, Massachusetts, USA, www.graphpad.com. Data are presented as mean ± standard deviation for continuous variables and as percentages for categorical. Ordinary one way ANOVA was used to identify differences in averages within disorders across indices and within an index across disorders. Results reported as a *p*-value with a significance cutoff <0.05.

## 3 Results

A total of 10 inborn errors of fatty acid metabolism were included in data collection and queried using Google Search Engine via search functions described in Table 1. For each disorder, the top 25 search results were analyzed for readability unless a paywall was encountered. Table 2 demonstrates the availability of the top 25 searches for each term. Searches with resulting material behind a paywall were not included in analysis.

**Table 1:** Internet search terms and associated acronyms of Inborn Errors of Metabolism.

Diseases Included in Analysis/Search Term	Acronym Used in Results Section
Medium-Chain Acyl-CoA Dehydrogenase Deficiency	“MCADD”
Primary Carnitine Deficiency/Carnitine Transporter Deficiency	“CTD”
Carnitine Palmitoyltransferase II Deficiency	“CPTD”
Very Long-Chain Acyl-CoA Dehydrogenase Deficiency	“VLCADD”
Trifunctional Protein Deficiency	“TPD”
Carnitine Palmitoyltransferase I Deficiency	“CPT1D”
Carnitine Acylcarnitine Translocase Deficiency	“CATD”
Multiple Acyl-CoA Dehydrogenase Deficiency	“MADD”
Hydroxyapatite Deposition Disease	“HADD”
Short-Chain Acyl-CoA Dehydrogenase Deficiency	“SCADD”

**Table 2:** Number of Top 25 searches included in analysis by inborn error of metabolism.

Inborn Error of Fatty Acid Metabolism	Number of Top 25 Searches Available (%)
Medium-Chain Acyl-CoA Dehydrogenase Deficiency	24 (96 %)
Primary Carnitine Deficiency/Carnitine Transporter Deficiency	23 (92 %)
Carnitine Palmitoyltransferase II Deficiency	23 (92 %)
Very Long-Chain Acyl-CoA Dehydrogenase Deficiency	23 (92 %)
Trifunctional Protein Deficiency	23 (92 %)
Carnitine Palmitoyltransferase I Deficiency	25 (100 %)
Carnitine Acylcarnitine Translocase Deficiency	24 (96 %)
Multiple Acyl-CoA Dehydrogenase Deficiency	20 (80 %)
Hydroxyapatite Deposition Disease	23 (92 %)
Short-Chain Acyl-CoA Dehydrogenase Deficiency	24 (96 %)

Average readability was calculated across indices for each disorder, detailed in Table 3 and Fig. 2. Briefly, SCADD demonstrated the lowest overall readability across indices at 11.64 (±1.90) and MADD was the highest overall at 13.32 (±1.70). Carnitine acylcarnitine translocase deficiency demonstrated the lowest individual index readability score of 9.61 (±2.34) using SMOG. MADD demonstrated the highest single index score of 15.08 (±1.30) using the Coleman-Liau equation. Comparison of the readability scores calculated by indices with an inborn error of fatty acid metabolism showed a consistent statistically significant difference. Interestingly, there was no statistically significant difference in the readability scores within a given index across disorders.

**Table 3:** Readability scores categorized by inborn error of metabolism.

Readability Scores						
Inborn Error of Fatty Acid Metabolism		Readability Scores Calculated by Index				
		*Flesch-Kincaid*	*Coleman-Liau*	*SMOG*	*Average of All*	
		Mean (±SD)	Mean (±SD)	Mean (±SD)	Mean (±SD)	p-value
**Medium-Chain Acyl-CoA Dehydrogenase Deficiency**						
		11.72 (3.18)	13.48 (2.89)	10.60 (2.61)	11.93 (1.45)	**0.0040**
**Creatine Transporter Deficiency**						
		12.61 (3.81)	14.62 (2.90)	11.33 (3.08)	12.85 (1.66)	**0.0056**
**Carnitine Palmitoyltransferase II Deficiency**						
		12.41 (3.31)	13.96 (2.39)	11.03 (2.79)	12.47 (1.46)	**0.0039**
**Very Long-Chain Acyl-CoA Dehydrogenase Deficiency**						
		11.78 (2.87)	13.88 (2.51)	10.37 (2.49)	12.01 (1.77)	**0.0001**
**Trifunctional Protein Deficiency**						
		11.70 (3.18)	14.07 (2.71)	10.27 (2.53)	12.01 (1.92)	**<0.0001**
**Carnitine Palmitoyltransferase I Deficiency**						
		11.42 (3.59)	13.67 (3.31)	9.91 (2.59)	11.67 (1.89)	**0.0004**
**Carnitine Acylcarnitine Translocase Deficiency**						
		11.89 (3.80)	14.75 (3.93)	9.61 (2.34)	12.08 (2.57)	**<0.0001**
**Multiple Acyl-CoA Dehydrogenase Deficiency**						
		13.18 (1.45)	15.08 (1.30)	11.69 (1.29)	13.32 (1.70)	**<0.0001**
**Hydroxyapatite Crystal Deposition Disease**						
		11.88 (2.56)	14.35 (2.47)	10.22 (1.98)	12.15 (2.08)	**<0.0001**
**Short-Chain Acyl-CoA Dehydrogenase Deficiency**						
		11.15 (3.14)	13.73 (2.78)	10.04 (2.43)	11.64 (1.90)	**<0.0001**
p-value		0.0629	0.6434	0.1226		

**Fig. 1:**
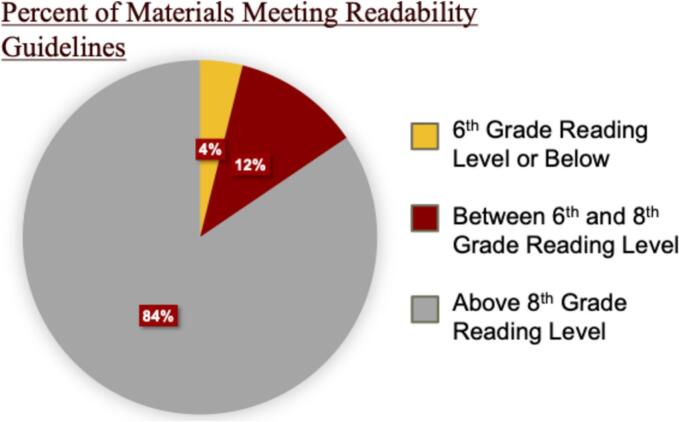
Percentage of Patient Facing Materials Meeting Recommended Grade Reading Levels.

Overall, of the inborn errors in fatty acid metabolism websites queried, 84 % demonstrated an average reading level greater than 8th grade. A total of 12 % had a calculated reading level between 6th and 8th grade, and 4 % showed a reading level < 6th grade (Fig. 1). A total of 3 or less publicly available resources per inborn error of metabolism showed a calculated readability level at or below either the NIH or AMA recommendation (Fig. 3).

**Fig. 2:**
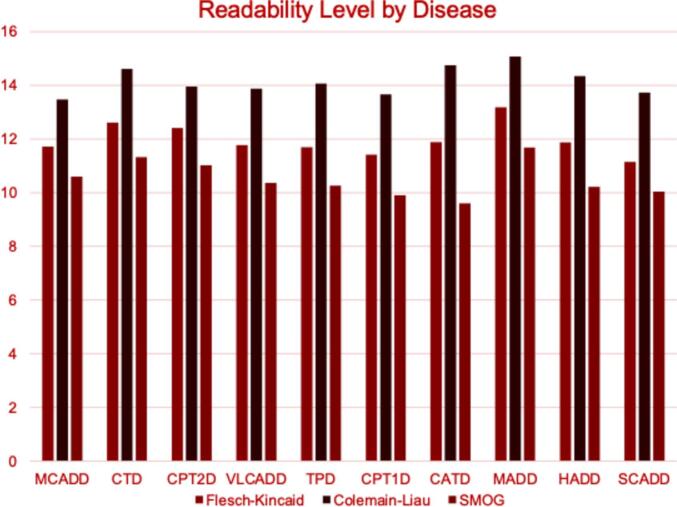
Stratification of Patient Facing Readability by Inborn Error of Metabolism.

**Fig. 3:**
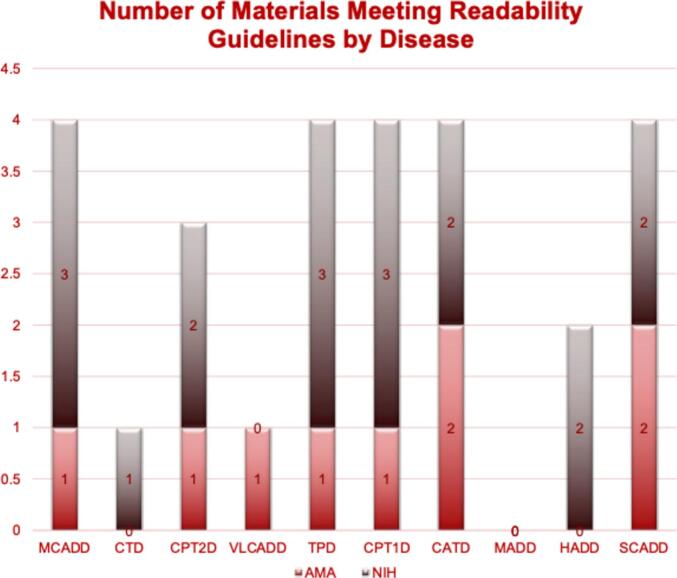
Distribution of Patient Facing Educational Materials Meeting Recommended Grade Reading Level by Inborn Error of Metabolism.

## 4 Discussion

The results of our study highlight a significant deficiency in the readability of online resources related to FAODs. Despite recommendations from the NIH and AMA that patient-facing materials be written at no higher than an eighth grade reading level, the majority of the analyzed resources exceeded this guideline. This discrepancy is concerning, given that many parents may rely heavily on online resources to understand and manage their child's condition.

The readability indices employed all revealed that the average reading levels of the materials analyzed were between 11th and 12th grade, suggesting that the information available to the public is written in a manner that may be too complex for a significant portion of the population to fully comprehend and engage with in an informed manner. The implications of this are far reaching, as parents with lower health literacy may struggle to make informed decisions or fully participate in their child's care, which risks compromising the therapeutic alliance and may lead to worse health outcomes.

While there was a significant variation in readability scores across different indices, no significant difference was found within a given index across the different FAODs. This consistency across disorders points to the complexity of online materials being a widespread issue, rather than one isolated to specific conditions. The few resources that did meet the NIH and AMA guidelines may be insufficient to serve the needs of the broader population, and are likely to be drowned out by the larger number of resources with a higher readability score. This underscores the urgent need for more accessible information.

There are a few limitations of our analysis of the readability of patient facing FAOD materials. First, our analysis was limited to the top 25 search results for each disorder which may preclude the true breadth of the literature. Secondly, the readability indices used, while previously validated, focus on the structural aspects of text, such as sentence length and word complexity. These indices do not account for other factors that can influence comprehension, like the use of medical jargon, cultural context, or the clarity of the content. Finally, our study was limited to English-language materials which limits the generalizability of our findings to the general population.

Our findings align with previous research indicating that much of the health information available online is written at a level too complex for the average reader. Given the increasing reliance on the internet for health information, especially among populations that may not have regular access to the healthcare system, it will be of continuing importance for publicly accessible healthcare resources to be revised closer to NIH and AMA guidelines.

## 5 Conclusion

Our study underscores the need for improving the readability of online resources. While our study focused on disorders of fatty acid oxidation, the need for improved readability is an ongoing concern that has been studied across many other medical disciplines. Aligning these and other resources with recommended reading levels, as set by the NIH and AMA, is an essential component of empowering the parents of children with FAODs and other rare childhood disorders to fully engage with the health of their children. Future efforts should focus on revising existing materials and developing new resources that are both accurate and easily understandable to ensure that all parents, regardless of their health literacy level, can access the information they need.

## CRediT authorship contribution statement

**Katelyn Sawyer:** Writing – review & editing, Writing – original draft, Visualization, Methodology, Investigation, Data curation, Conceptualization. **William Miller:** Writing – review & editing, Methodology, Investigation, Formal analysis, Conceptualization. **Courtney Popp:** Conceptualization. **Chloe Strege:** Conceptualization. **Cindy Eide:** Writing – review & editing, Supervision, Project administration, Conceptualization. **Jakub Tolar:** Supervision, Project administration, Funding acquisition.

## Declaration of competing interest

None.

## Data Availability

Data will be made available on request.

## Appendix A Supplementary data

Supplementary material: Internet Patient Facing Readability Scores by Individual Site Image 1
